# Research on Initial Alignment and Self-Calibration of Rotary Strapdown Inertial Navigation Systems

**DOI:** 10.3390/s150203154

**Published:** 2015-01-30

**Authors:** Wei Gao, Ya Zhang, Jianguo Wang

**Affiliations:** 1 College of Automation, Harbin Engineering University, Harbin 150001, China; E-Mail: gaow@hrbeu.edu.cn; 2 School of Electrical Engineering and Automation, Harbin Institute of Technology, Harbin 150001, China; 3 Department of Earth and Space and Engineering, York University, Toronto, ON M3J 1P3, Canada; E-Mail: jgwang@yorku.ca

**Keywords:** strapdown inertial navigation system (SINS), rotating SINS, dual-axis rotary, initial alignment and self-calibration, observability

## Abstract

The errors of inertial sensors affect the navigation accuracy of the strapdown inertial navigation system (SINS) and are accumulated over time in nature. In order to continuously maintain the high navigation accuracy of vehicles for a long time period, an initial alignment and self-calibration is necessary after the SINS starts. Additionally, the observability analysis is one of the key techniques during the initial alignment and self-calibration process. For marine systems, the observability of inertial sensor errors is extremely low, as their motion states are always slow. Therefore, studying the rotating SINS is urgent. Since traditional analysis methods have their limitations, the global observation analysis method was used in this paper. On the basis of this method, the relationship between the observability and the kinestate of the rotating SINS has been established. After the discussion about the factors that affect the observability in detail, the design principle of the initial alignment and self-calibration rotating scheme, which is appropriate for marine systems, id proposed. With the proposed principle, a novel initial alignment and self-calibration method, named the eight-position rotating scheme, is designed. Simulations and experiments are carried out to verify its performance. The results have shown that compared with other rotating schemes and the static state, the estimated accuracy of the eight-position scheme rotating about axes *x* and *y* was the best, and the position error was significantly reduced with this new rotating scheme. The feasibility and effectiveness of the proposed design principle and the rotating scheme were verified.

## 1. Introduction

In modern marine navigation, the strapdown inertial navigation system (SINS) is widely used, due to its advantages of being more compact and autonomous [1–4]. However, the successive starting error of inertial sensors is one of the most major factors that will affect the system accuracy. Therefore, to maintain high navigation accuracy for a long time period, it is necessary to perform on-line calibration after the SINS starts [5–8]. The core of the SINS on-line calibration is that changing the motion states of the inertial measurement unit (IMU), including the angular motion and the linear motion, can significantly improve the system observability, and then, the optimal estimation of the inertial sensor errors can be obtained [9–11]. For high-speed carriers, such as aircraft, it is obviously easy to change the motion states. However, for sailing ships on the sea surface, during the mooring, the maneuver can be overlooked by approximation; even during sailing, the motor is still unobvious, as the acceleration, de-acceleration and turning of ships are all extremely slow. Therefore, for vessels, it is not easy to change the spatial position of the IMU. In order to deal with the above-mentioned problem, the dual-axial rotating SINS emerged. In the rotating SINS, according to specific rotary schemes, the IMU spatial position can be changed by utilizing a rotating mechanism that has two degrees of freedom, increasing the observability of the inertial sensor errors dramatically [10,12,13].

As we all know, the self-calibration of the rotary SINS can be seen as a system optimal estimation using the Kalman filter, so the observability analysis of the SINS is required before the filter design [14–16]. The traditional observability analysis methods, such as the piece-wise constant system (PWCS) and the singular value decomposition (SVD), are the most commonly used [17–19]. A time-varying system can be approximated by the PWCS method with little loss of the characteristics of the time response. The SVD method is widely used in the SINS, because the filtering process is not needed at all before the observability analysis. However, both of these methods have their limitations. The PWCS method is only able to get the number of the observed system states, while the specific observability degree cannot be determined. For the incompletely observed systems in which the states are coupled, the SVD method cannot explicitly explain the coupling [20–22].

To perfect the above methods' deficiencies, numerous researchers and scholars are dedicating themselves to further researching and improving the methods of observability analysis. One of the most notably modified methods is the global observability analysis method based on the original nonlinear models, proposed in 2011 by Wu *et al.* [9,23,24]. It should be noted that in most past research, conservative observability concepts, e.g., local observability and linear observability, have extensively been used to locally characterize the estimability. However, the global observability analysis can provide us with comprehensive instructions on whether the estimation is feasible under a given condition or not, as well as how to achieve the state estimates, e.g., by resorting to vehicle maneuvers. Additionally, the global observability analysis method can be used in nonlinear systems. Directly starting from the definition of the observability, this method takes the nonlinear models of the SINS as the basic equations of the observability analysis. After the basic equations are solved, the global observability of the SINS can be analyzed. The global observability analysis method can conduct the observability analysis of the SINS simply, directly and effectively, by avoiding the drawbacks of the traditional methods.

A self-calibration method for dual-axis rotation-modulating INS was introduced in [25], and a calibration rotation sequence was designed. Sun *et al.* [26] proposed an eight-position calibration method, which can achieve calibration inertial sensor errors when the attitudes were unknown. However, the accuracy of these existing methods was not high, so we proposed a new rotation method for self-calibration and initial alignment.

Based on the global observability analysis, the relationship between resident positions and rotations of the IMU and the relationship between the observability and the kinestate of the rotating SINS have been established in this paper. Accordingly, the design principle of the initial alignment and self-calibration rotating scheme for the SINS was proposed. Besides, by taking the proposed design principle into account, an optimum rotating scheme was designed in this paper. The numerical simulations and experiments showed that the proposed rotary scheme was superior to others, and the position error can be reduced significantly with this new rotary scheme. The rest of the paper is organized as follows. The design principle of the rotating scheme of the SINS is described in Section 2. Section 3 proposes a new rotating scheme based on the design principle. Numerical examples and experiments along with specific analysis are given in Section 4 and Section 5, respectively. Section 6 concludes this manuscript.

## 2. Design Principle of the Rotating Scheme

With the design of the initial alignment and self-calibration of the dual-axis rotary SINS, the key is to determine resident positions and to choose rotating axes [9], which will be discussed respectively in this section.

### 2.1. Determination of Resident Positions

Assume that inertial sensor errors are just constant, then the output of the gyroscopes can be expressed as:

(1) $${\tilde{\omega }}_{ib}^{b}=\omega_{ib}^{b}+ɛ$$

wherein *i* indicates the inertial coordinate system, while b indicates the body coordinate system; and vectors ${\tilde{\omega }}_{ib}^{b}$, $\omega_{ib}^{b}$ and ***ε*** are the raw output, the theoretical output and the constant drift of the gyroscopes, respectively.

From Equation (1), we can get:

(2) $$\left|{\tilde{\omega }}_{ib}^{b}-ɛ\right|=\left|\omega_{ib}^{b}\right|=\Omega$$

Equation (2) can be explained as a spherical surface, whose center is the endpoint of vector ${\tilde{\omega }}_{ib}^{b}$ with the radius **Ω**, while ***ε*** can be explained as a moving point on this spherical surface. Therefore, the observability of ***ε*** is about determining the unique point on this spherical surface.

If there are three spherical surfaces intersected with each other, the number of the intersection points is two. Moreover, these two intersection points are symmetrical about the plane formed by three sphere centers. Therefore, it is not enough if there are only three constraints. If we want to determine the unique point further, another spherical surface whose center is not in the plane should be introduced [13]. Hence, ***ε*** can be shown as:

(3) $$ɛ={\left(\begin{array}{cc} {\left[\begin{array}{c} 2{\left[{\left({\tilde{\omega }}_{ib}^{b}\right)}_{1}-{\left({\tilde{\omega }}_{ib}^{b}\right)}_{2}\right]}^{T}\\ 2{\left[{\left({\tilde{\omega }}_{ib}^{b}\right)}_{1}-{\left({\tilde{\omega }}_{ib}^{b}\right)}_{3}\right]}^{T}\\ 2{\left[{\left({\tilde{\omega }}_{ib}^{b}\right)}_{1}-{\left({\tilde{\omega }}_{ib}^{b}\right)}_{4}\right]}^{T} \end{array}\right]}^{T} & \left[\begin{array}{c} 2{\left[{\left({\tilde{\omega }}_{ib}^{b}\right)}_{1}-{\left({\tilde{\omega }}_{ib}^{b}\right)}_{2}\right]}^{T}\\ 2{\left[{\left({\tilde{\omega }}_{ib}^{b}\right)}_{1}-{\left({\tilde{\omega }}_{ib}^{b}\right)}_{3}\right]}^{T}\\ 2{\left[{\left({\tilde{\omega }}_{ib}^{b}\right)}_{1}-{\left({\tilde{\omega }}_{ib}^{b}\right)}_{4}\right]}^{T} \end{array}\right] \end{array}\right)}^{-1}\;{\left[\begin{array}{c} 2{\left[{\left({\tilde{\omega }}_{ib}^{b}\right)}_{1}-{\left({\tilde{\omega }}_{ib}^{b}\right)}_{2}\right]}^{T}\\ 2{\left[{\left({\tilde{\omega }}_{ib}^{b}\right)}_{1}-{\left({\tilde{\omega }}_{ib}^{b}\right)}_{3}\right]}^{T}\\ 2{\left[{\left({\tilde{\omega }}_{ib}^{b}\right)}_{1}-{\left({\tilde{\omega }}_{ib}^{b}\right)}_{4}\right]}^{T} \end{array}\right]}^{T}\left[\begin{array}{c} {\left|{\left({\tilde{\omega }}_{ib}^{b}\right)}_{1}\right|}^{2}-{\left|{\left({\tilde{\omega }}_{ib}^{b}\right)}_{2}\right|}^{2}\\ {\left|{\left({\tilde{\omega }}_{ib}^{b}\right)}_{1}\right|}^{2}-{\left|{\left({\tilde{\omega }}_{ib}^{b}\right)}_{3}\right|}^{2}\\ {\left|{\left({\tilde{\omega }}_{ib}^{b}\right)}_{1}\right|}^{2}-{\left|{\left({\tilde{\omega }}_{ib}^{b}\right)}_{4}\right|}^{2} \end{array}\right]$$

wherein ${\left({\tilde{\omega }}_{ib}^{b}\right)}_{j}$ is the output when the gyroscopes are in the *j*—*th* (*j* = 1, 2,…, *n*) position.

Homoplastically, the zero bias of the accelerometers **∇** can also be determined uniquely. The output vector of the accelerometers is:

(4) $${\tilde{f}}_{ib}^{b}=f_{ib}^{b}+\nabla$$

wherein vectors ${\tilde{f}}_{ib}^{b}$ and $f_{ib}^{b}$ are the raw output and the theoretical output of the accelerometers, respectively.

In a similar way to ***ε***, **∇** can be deduced as:

(5) $$\nabla ={\left(\begin{array}{cc} {\left[\begin{array}{c} 2{\left[{\left({\tilde{f}}_{ib}^{b}\right)}_{1}-{\left({\tilde{f}}_{ib}^{b}\right)}_{2}\right]}^{T}\\ 2{\left[{\left({\tilde{f}}_{ib}^{b}\right)}_{1}-{\left({\tilde{f}}_{ib}^{b}\right)}_{3}\right]}^{T}\\ 2{\left[{\left({\tilde{f}}_{ib}^{b}\right)}_{1}-{\left({\tilde{f}}_{ib}^{b}\right)}_{4}\right]}^{T} \end{array}\right]}^{T} & \left[\begin{array}{c} 2{\left[{\left({\tilde{f}}_{ib}^{b}\right)}_{1}-{\left({\tilde{f}}_{ib}^{b}\right)}_{2}\right]}^{T}\\ 2{\left[{\left({\tilde{f}}_{ib}^{b}\right)}_{1}-{\left({\tilde{f}}_{ib}^{b}\right)}_{3}\right]}^{T}\\ 2{\left[{\left({\tilde{f}}_{ib}^{b}\right)}_{1}-{\left({\tilde{f}}_{ib}^{b}\right)}_{4}\right]}^{T} \end{array}\right] \end{array}\right)}^{-1}\begin{array}{cc} {\left[\begin{array}{c} 2{\left[{\left({\tilde{f}}_{ib}^{b}\right)}_{1}-{\left({\tilde{f}}_{ib}^{b}\right)}_{2}\right]}^{T}\\ 2{\left[{\left({\tilde{f}}_{ib}^{b}\right)}_{1}-{\left({\tilde{f}}_{ib}^{b}\right)}_{3}\right]}^{T}\\ 2{\left[{\left({\tilde{f}}_{ib}^{b}\right)}_{1}-{\left({\tilde{f}}_{ib}^{b}\right)}_{4}\right]}^{T} \end{array}\right]}^{T} & \left[\begin{array}{c} {\left|{\left({\tilde{f}}_{ib}^{b}\right)}_{1}\right|}^{2}-{\left|{\left({\tilde{f}}_{ib}^{b}\right)}_{2}\right|}^{2}\\ {\left|{\left({\tilde{f}}_{ib}^{b}\right)}_{1}\right|}^{2}-{\left|{\left({\tilde{f}}_{ib}^{b}\right)}_{3}\right|}^{2}\\ {\left|{\left({\tilde{f}}_{ib}^{b}\right)}_{1}\right|}^{2}-{\left|{\left({\tilde{f}}_{ib}^{b}\right)}_{4}\right|}^{2} \end{array}\right] \end{array}$$

wherein ${\left({\tilde{f}}_{ib}^{b}\right)}_{j}$ is the accelerometer output at the *j*—*th* (*j* = 1, 2,…, *n*) position.

Based on the above analysis, we can learn how to determine the resident positions in principle. That is, the IMU should stay at four different positions at least. Only if this condition is satisfied can the gyroscope drifts and the accelerometer biases be observed completely.

### 2.2. The Choices of Rotary Axes

According to the fundamentals of the SINS, the basic equations of the global observability analysis are as follows [24]:

(6) $$\{\begin{array}{c} {\dot{C}}_{b}^{n}=C_{b}^{n}\left[\left({\tilde{\omega }}_{ib}^{b}-ɛ\right)\times \right]-\left[\left(\omega_{ie}^{n}+\omega_{en}^{n}\right)\times \right]C_{b}^{n}\\ {\dot{\upsilon }}^{n}=C_{b}^{n}\left({\tilde{f}}_{ib}^{b}-\nabla \right)-\left(2\omega_{ie}^{n}+\omega_{en}^{n}\right)\times \upsilon^{n}+g^{n} \end{array}$$

wherein $C_{b}^{n}$ denotes the direction cosine matrix from the *b* coordinate system to the navigation coordinate system (*n*); vectors ${\tilde{f}}_{ib}^{b}$ and ${\tilde{\omega }}_{ib}^{b}$ are the raw outputs of the accelerometers and the gyroscopes, respectively; $\omega_{ie}^{n}$ indicates the Earth's rotating angular rate vector; $\omega_{en}^{n}$ is the rotating angular rate vector in the *n* coordinate system relative to the Earth coordinate system (e); ***g****^n^* denotes the gravity vector in the *n* coordinate system; ***υ**^n^* is the transporter's velocity of the *n* coordinate system and is zero when the transporter is in the stationary base.

Considering that in the stationary base, the IMU is rotating at a uniform velocity around the sensitive axis, Equation (6) can be rewritten as follows:

(7) $$\{\begin{array}{c} {\tilde{\omega }}_{ib}^{b}=\omega_{nb}^{b}+C_{n}^{b}\omega_{ie}^{n}+ɛ\\ {\tilde{f}}_{ib}^{b}=C_{n}^{b}g^{n}+\nabla \end{array}$$

wherein $\omega_{nb}^{b}$ is the rotating angular rate vector in the *b* coordinate system relative to the *n* coordinate system.

The differentiation of Equation (7) gives:

(8) $$\{\begin{array}{c} {\dot{\tilde{\omega }}}_{ib}^{b}={\dot{\omega }}_{nb}^{b}+{\dot{C}}_{n}^{b}\omega_{ie}^{n}={\dot{C}}_{n}^{b}\omega_{ie}^{n}=-\omega_{nb}^{b}\times \left(C_{n}^{b}\omega_{ie}^{n}\right)\\ {\dot{\tilde{f}}}_{ib}^{b}=-{\dot{C}}_{n}^{b}g^{n}=\omega_{nb}^{b}\left(C_{n}^{b}g^{n}\right) \end{array}$$

Further, the differentiation of Equation (8) yields:

(9) $$\{\begin{array}{c} {\ddot{\tilde{\omega }}}_{ib}^{b}=-\omega_{nb}^{b}\times \left(-\omega_{nb}^{b}\times C_{n}^{b}\right)=-\omega_{nb}^{b}\times {\dot{\tilde{\omega }}}_{ib}^{b}\\ {\ddot{\tilde{f}}}^{b}=\omega_{nb}^{b}\times \left({\dot{C}}_{n}^{b}g^{n}\right)=-\omega_{nb}^{b}\times {\dot{\tilde{f}}}_{ib}^{b} \end{array}$$

Since ${\dot{\tilde{\omega }}}_{ib}^{b}=-\omega_{nb}^{b}\times \left(C_{n}^{b}\omega_{ie}^{n}\right)$ and the property of the vector [9], we know that $C_{n}^{b}\omega_{ie}^{n}$ is a vector in the plane, which is formed by vectors $\omega_{nb}^{b}$ and $\omega_{nb}^{b}\times {\dot{\tilde{\omega }}}_{ib}^{b}$.

Therefore, $C_{n}^{b}\omega_{ie}^{n}$ can be expressed as:

(10) $$C_{n}^{b}\omega_{ie}^{n}=k_{1}\omega_{nb}^{b}+k_{2}\;\left(\omega_{nb}^{b}\times {\dot{\tilde{\omega }}}_{ib}^{b}\right)$$

wherein both *k*_1_ and *k*_2_ are constant.

Furthermore, due to:

(11) $$\omega_{nb}^{b}⊥\left(\omega_{nb}^{b}\times {\dot{\tilde{\omega }}}_{ib}^{b}\right)$$

we can get:

(12) $$\omega_{nb}^{b}\;\cdot \;\left(\omega_{nb}^{b}\times {\dot{\tilde{\omega }}}_{ib}^{b}\right)=0$$

Furthermore, we can obtain:

(13) $$k_{2}=-\frac{1}{{\left|\omega_{nb}^{b}\right|}^{2}}$$

After Equation (10) and:

(14) $${\left|C_{n}^{b}\omega_{ie}^{n}\right|}^{2}=\Omega^{2}$$

We have:

(15) $${\left[k_{1}\omega_{nb}^{b}+k_{2}\;\left(\omega_{nb}^{b}\times {\dot{\tilde{\omega }}}_{ib}^{b}\right)\right]}^{2}=\Omega^{2}$$

By expanding the left side of Equation (15), the equation can be expressed as follows:

(16) $${k_{1}}^{2}{\left|\omega_{nb}^{b}\right|}^{2}+2k_{1}k_{2}\omega_{nb}^{b}\left(\omega_{nb}^{b}\times {\dot{\tilde{\omega }}}_{ib}^{b}\right)+{k_{2}}^{2}\;{\left(\omega_{nb}^{b}\times {\dot{\tilde{\omega }}}_{ib}^{b}\right)}^{2}=\Omega^{2}$$

Since:

(17) $$\omega_{nb}^{b}⊥\left(\omega_{nb}^{b}\times {\dot{\tilde{\omega }}}_{ib}^{b}\right)$$

Equation (16) is simplified to:

(18) $${k_{1}}^{2}{\left|\omega_{nb}^{b}\right|}^{2}+{k_{2}}^{2}{\left|\omega_{nb}^{b}\right|}^{2}\;{\left|{\dot{\tilde{\omega }}}_{ib}^{b}\right|}^{2}=\Omega^{2}$$

From Equations (13) and (18), *k*_1_ is as follows:

(19) $$k_{1}=\pm \frac{\sqrt{{\left|\omega_{nb}^{b}\right|}^{2}\Omega^{2}-{\left|{\dot{\tilde{\omega }}}_{ib}^{b}\right|}^{2}}}{{\left|\omega_{nb}^{b}\right|}^{2}}$$

According to Equations (10), (13) and (19), the following equation can easily be deduced:

(20) $$C_{n}^{b}\omega_{ie}^{n}=\pm \frac{\sqrt{{\left|\omega_{nb}^{b}\right|}^{2}\Omega^{2}-{\left|{\dot{\tilde{\omega }}}_{ib}^{b}\right|}^{2}}}{{\left|\omega_{nb}^{b}\right|}^{2}}\omega_{nb}^{b}-\frac{1}{{\left|\omega_{nb}^{b}\right|}^{2}}\omega_{nb}^{b}\times {\dot{\tilde{\omega }}}_{ib}^{b}$$

Further deduction of Equation (20) gives:

(21) $$C_{n}^{b}\omega_{ie}^{n}=C_{n}^{b}\omega_{ie}^{n}-\frac{\omega_{nb}^{b}\left(C_{n}^{b}\omega_{ie}\right)}{{\left|\omega_{nb}^{b}\right|}^{2}}\omega_{nb}^{b}\pm \frac{\omega_{nb}^{b}\left(C_{n}^{b}\omega_{ie}\right)}{{\left|\omega_{nb}^{b}\right|}^{2}}\omega_{nb}^{b}$$

Similarly, $C_{n}^{b}g^{n}$ can also be obtained as follows:

(22) $$C_{n}^{b}g^{n}=C_{n}^{b}g^{n}-\frac{\omega_{nb}^{b}\left(C_{n}^{b}g^{n}\right)}{{\left|\omega_{nb}^{b}\right|}^{2}}\omega_{nb}^{b}\pm \frac{\omega_{nb}^{b}\left(C_{n}^{b}g^{n}\right)}{{\left|\omega_{nb}^{b}\right|}^{2}}\omega_{nb}^{b}$$

From Equations (21) and (22), $C_{n}^{b}\omega_{ie}$ and $C_{n}^{b}g^{n}$ can obtain two solutions:

(23) $$\{\begin{array}{c} {\left(C_{n}^{b}\omega_{ie}^{n}\right)}_{1}=C_{n}^{b}\omega_{ie}^{n}\\ {\left(C_{n}^{b}\omega_{ie}^{n}\right)}_{2}=C_{n}^{b}\omega_{ie}^{n}-2\frac{\omega_{nb}^{b}\left(C_{n}^{b}\omega_{ie}^{n}\right)}{{\left|\omega_{nb}^{b}\right|}^{2}}\omega_{nb}^{b} \end{array}$$

(24) $$\{\begin{array}{c} {\left(C_{n}^{b}g^{n}\right)}_{1}=C_{n}^{b}g^{n}\\ {\left(C_{n}^{b}g^{n}\right)}_{2}=C_{n}^{b}g^{n}-2\frac{\omega_{nb}^{b}\left(C_{n}^{b}g^{n}\right)}{{\left|\omega_{nb}^{b}\right|}^{2}}\omega_{nb}^{b} \end{array}$$

It is easy to know that only when each of $C_{n}^{b}\omega_{ie}^{n}$ and $C_{n}^{b}g^{n}$ has one solution uniquely, the ***ε*** and **∇** can be absolutely observed. Therefore, the situation that each of $C_{n}^{b}\omega_{ie}^{n}$ and $C_{n}^{b}g^{n}$ has two solutions is not acceptable. However, $\frac{\omega_{nb}^{b}\left(C_{n}^{b}\omega_{ie}^{n}\right)}{|\omega_{nb}^{b}{|}^{2}}\omega_{nb}^{b}$ and $\frac{\omega_{nb}^{b}\left(C_{n}^{b}g^{n}\right)}{|\omega_{nb}^{b}{|}^{2}}\omega_{nb}^{b}$ can be seen as corresponding errors, while $C_{n}^{b}\omega_{ie}^{n}$ and $C_{n}^{b}g^{n}$ as true values. Then, only if the errors are zeros, the solutions are unique and ***ε*** and **∇** are absolutely observed.

When $\omega_{nb}^{b}⊥\left(C_{n}^{b}\omega_{ie}^{n}\right)$, that means, when the rotating direction of the IMU is perpendicular to the Earth's rotary axis, the value of $\frac{\omega_{nb}^{b}\left(C_{n}^{b}\omega_{ie}^{n}\right)}{|\omega_{nb}^{b}{|}^{2}}\omega_{nb}^{b}$ is zero, and in this condition, ***ε*** is absolutely observed. When $\omega_{nb}^{b}⊥\left(C_{n}^{b}g^{n}\right)$, that means, when the rotating direction of the IMU is on the horizontal plane, $\frac{\omega_{nb}^{b}\left(C_{n}^{b}g^{n}\right)}{|\omega_{nb}^{b}{|}^{2}}\omega_{nb}^{b}$ becomes zero, so that **∇** is absolutely observed.

From the above analysis, we can get the choosing principle of the rotary axis in the rotary SINS clearly: when the rotary axes are horizontal axes, the gyroscope drifts and the accelerometer biases are both absolutely observed.

## 3. New Initial Alignment and Self-Calibration Rotating Scheme

**Figure 1.**
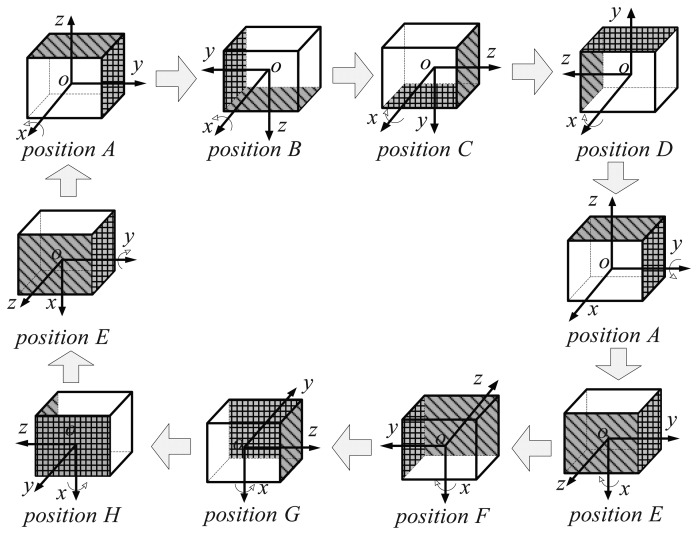
The eight-position rotation scheme: The proposed initial alignment and self-calibration method in dual-axis rotary SINS with eight different positions in which to reside and rotating about the *x* axis and *y* axis.

From the analysis in Section 2, the gyroscope drifts, and the accelerometer biases and the misalignment angles are all absolutely observed when the IMU alternately rotates about two horizontal axes and resides at four different positions at least. Therefore, to ensure that all of inertial sensor errors can completely be observed and to minimize the rotation number, a novel eight-position rotating scheme rotating about two horizontal axes was proposed here based on the above rotary principle. In this scheme, the IMU is in rotation and steady alternately. There is a small static state that keeps *t_stop_* after each rotation, wherein *t_stop_* is the residence time at each position. The rotation order in the new scheme is illustrated in Figure 1.

The IMU rotation starts from Position A, and the detailed explanation of this new rotating scheme is as follows:

A → B: rotate counterclockwise 180° about the *x* axis from Position A to Position B;
B → C: rotate counterclockwise 90° about the *x* axis from Position B to Position C;
C → D: rotate clockwise 180° about the *x* axis from Position C to Position D;
D → A: rotate clockwise 90° about the *x* axis from Position D to Position A;
A → E: rotate counterclockwise 90° about the *y* axis from Position A to Position E;
E → F: rotate counterclockwise 180° about the *x* axis from Position E to Position F;
F → G: rotate counterclockwise 90° about the *x* axis from Position F to Position G;
G → H: rotate clockwise 180° about the *x* axis from Position G to Position H;
H → E: rotate clockwise 90° about the *x* axis from Position H to Position E;
E → A: rotate clockwise 90° about the *y* axis from Position E to Position A.

In this novel rotation scheme, the IMU rotates about two horizontal axes (*x* axis and *y* axis) and resides at eight different positions to satisfy the design principle introduced in the previous section.

## 4. Simulations and Results

**Figure 2.**
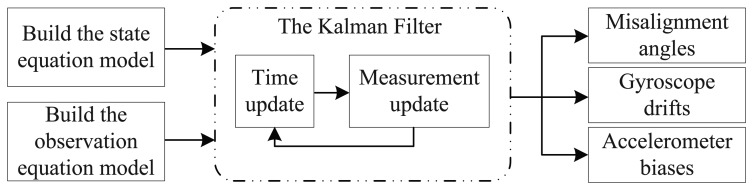
The block diagram of the Kalman filter.

In this section, simulations were introduced to check the feasibility of the proposed rotating scheme. From the discussions in previous sections, the initial alignment and self-calibration of the SINS can be seen as a system optimal estimation based on the Kalman filter, and the block diagram of the Kalman filter is presented as in Figure 2.

Thus, filtering equations of the SINS should first be established. The state equation of the SINS is symbolically as follows:

(25) $$\dot{X}\left(t\right)\;=F\left(t\right)\;X\left(t\right)\;+G\left(t\right)\;W\left(t\right)\;$$

wherein ***X***(*t*) is the state error vector at *t*; ***F***(*t*) is the state-transition matrix; ***W***(*t*) and ***G***(*t*) are the noise vector and the associated coefficient matrix, respectively.

The state vector and the noise vector are defined as follows:

(26) $$X\left(t\right)={\left[\delta L\;\delta \lambda \;\delta \upsilon_{E}\;\delta \upsilon_{N}\;\varphi_{x}\;\varphi_{y}\;\varphi_{z}\;\nabla_{x}\;\nabla_{y}\;\nabla_{z}\;{ɛ}_{x}\;{ɛ}_{y}\;{ɛ}_{z}\right]}^{T}$$

(27) $$W\left(t\right)\;={\left[\omega_{\nabla_{x}}\;\omega_{\nabla_{y}}\;\omega_{\nabla_{z}}\;\omega_{{ɛ}_{x}}\;\omega_{{ɛ}_{y}}\;\omega_{{ɛ}_{z}}\right]}^{T}$$

wherein *δL* and *δλ* are the errors of the longitude and the latitude, respectively; *δυ_E_* and *δυ_N_* are the east and north velocity errors; the misalignment angles between the calculated geographical coordinate system and the true geographical coordinate system are denoted by *φ_x_*, *φ_y_* and *φ_z_*; ∇*_x_*, ∇*_y_* and ∇*_z_* are the accelerometer biases in the *x*, *y* and *z* directions, respectively; *ω*∇*_x_*, *ω*∇*_y_* and *ω*∇*_z_* are the corresponding noises of the accelerometers; *ε_x_*, *ε_y_* and *ε_z_* are the gyroscope drifts in the *x*, *y* and *z* directions, respectively; *ω_εx_*, *ω_εy_* and *ω_ε_*_z_ are the corresponding noises of the gyroscopes.

Let us take the velocity errors as the measurement vector; the measurement equation is as follows:

(28) Z(t)=H(t)X(t)+V(t)

wherein ***Z*** is the measurement vector at *t* and ***H*** and ***V*** are the measurement design matrix and the measurement noise, respectively.

The matrix ***H*** is given as:

(29) $$H\left(t\right)\;=\left[0_{2\times 2}\;I_{2\times 2}\;0_{2\times 9}\right]$$

**Figure 3.**
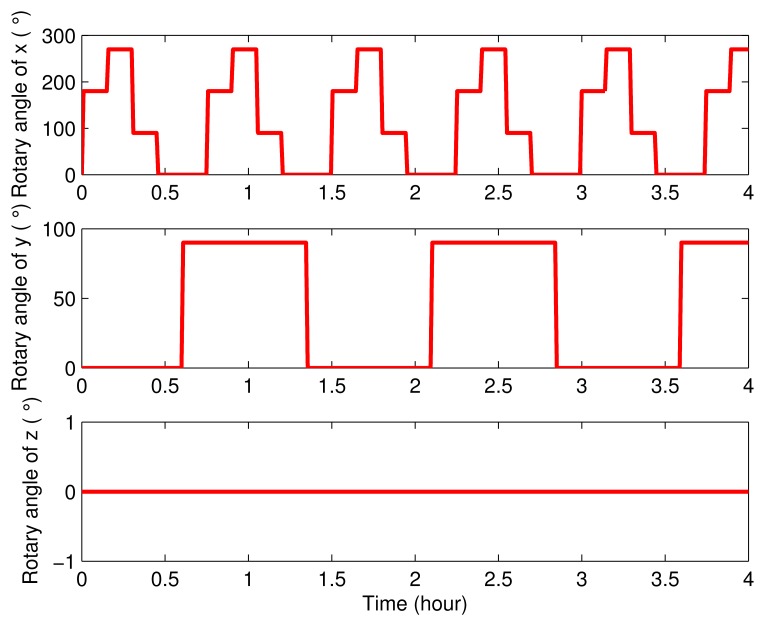
The rotary angle model of the proposed rotary scheme, which was rotating about the *x* axis and *y* axis.

**Figure 4.**
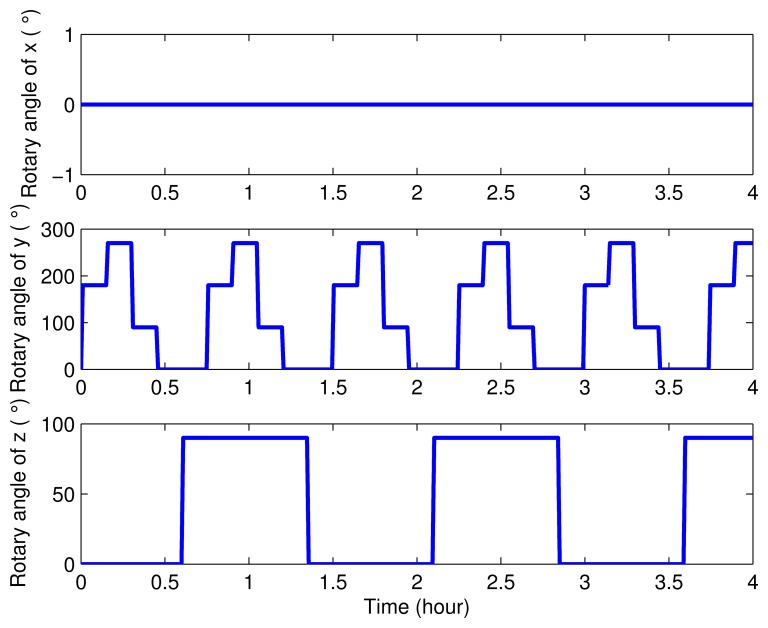
The rotary angle model of the proposed rotary scheme, which was rotating about the *y* axis and *z* axis.

Next, simulations were carried out to verify the effectiveness and superiority of the eight-position rotating scheme proposed in this paper. In order to make a better illustration, three groups of simulations and their results were compared below. In one simulation, the SINS was set as stationary. Contrarily, the IMU was set as rotary in the other two simulations. The only difference between these two simulations is in their rotating axes: the first one was rotating about the *x* axis and *y* axis proposed in this paper, while the second one was rotating about the *y* axis and *z* axis proposed in [26]. The settings of the rotary angles in these two rotary simulations are described in Figures 3 and 4.

**Figure 5.**
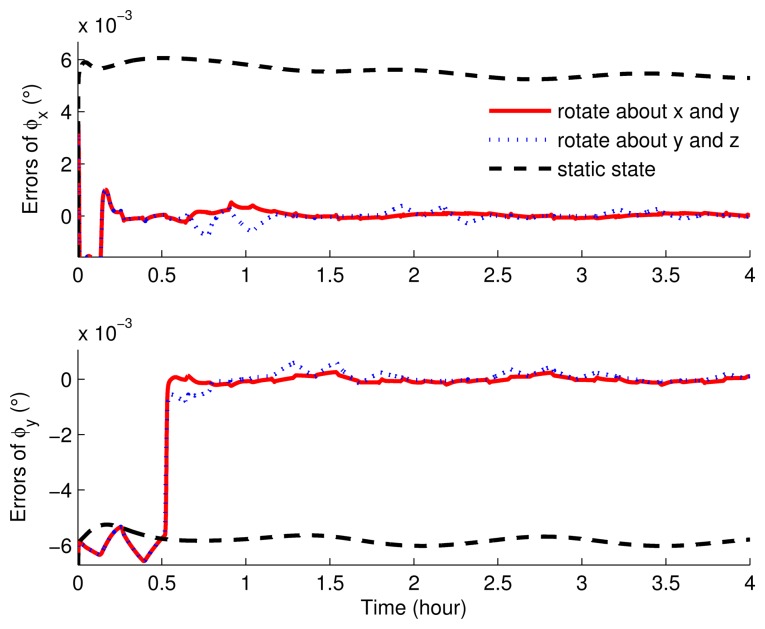
The estimated errors of the horizontal misalignment angles of rotation and static simulations.

**Figure 6.**
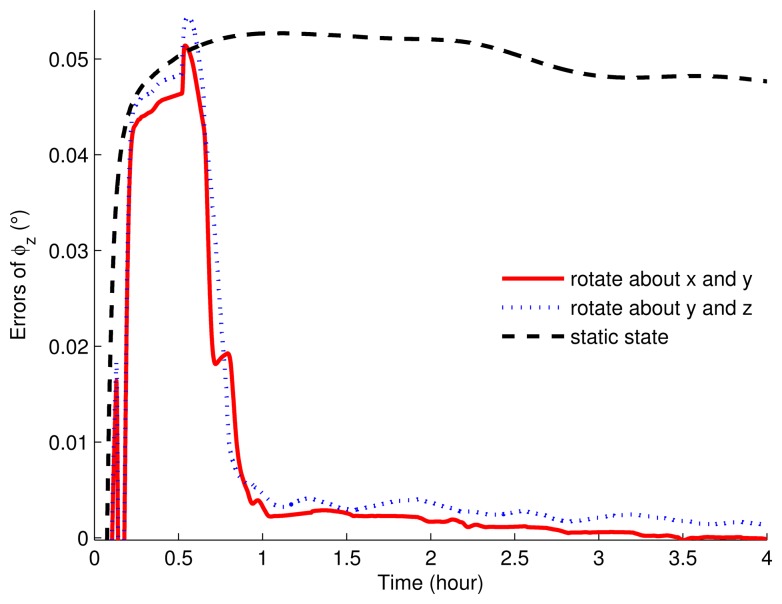
The estimated errors of the azimuth misalignment angles of rotation and static simulations.

**Figure 7.**
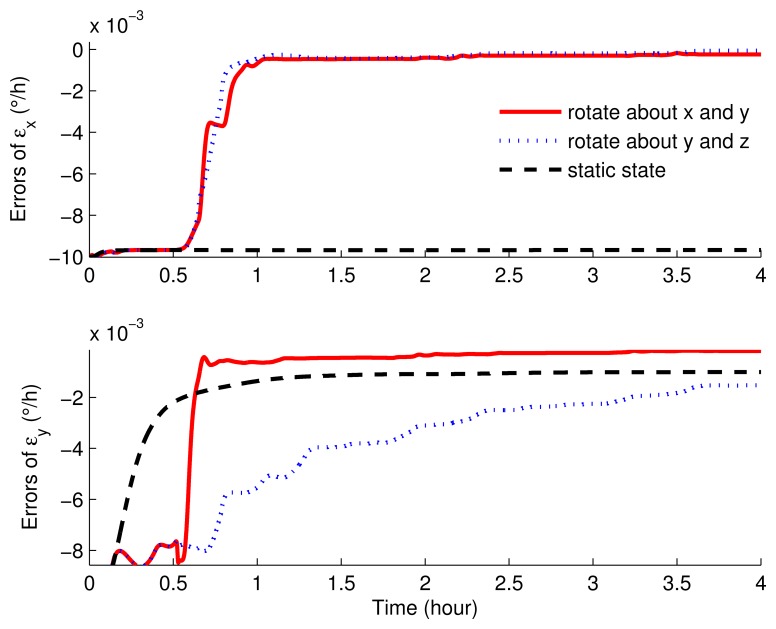
The estimated errors of the horizontal gyroscope drifts of the rotation and static simulations.

**Figure 8.**
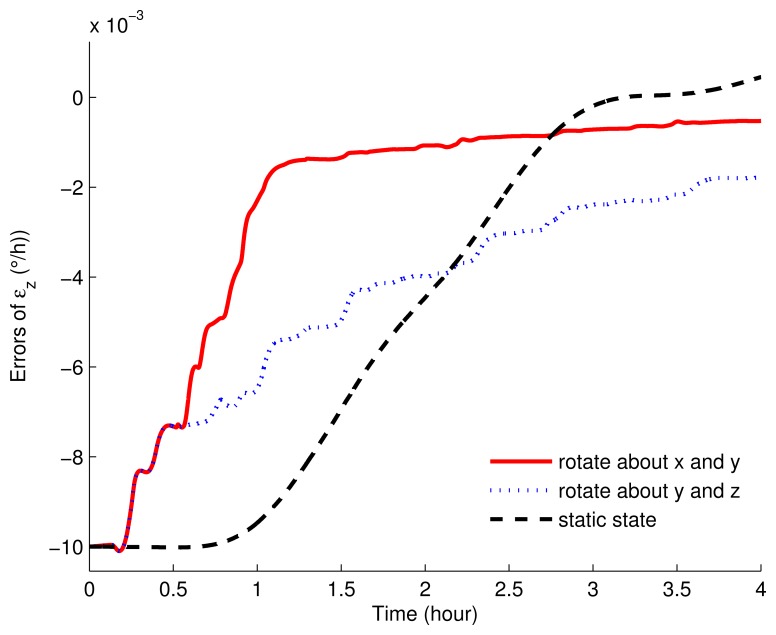
The estimated errors of the azimuth gyroscope drifts of the rotation and static simulations.

**Figure 9.**
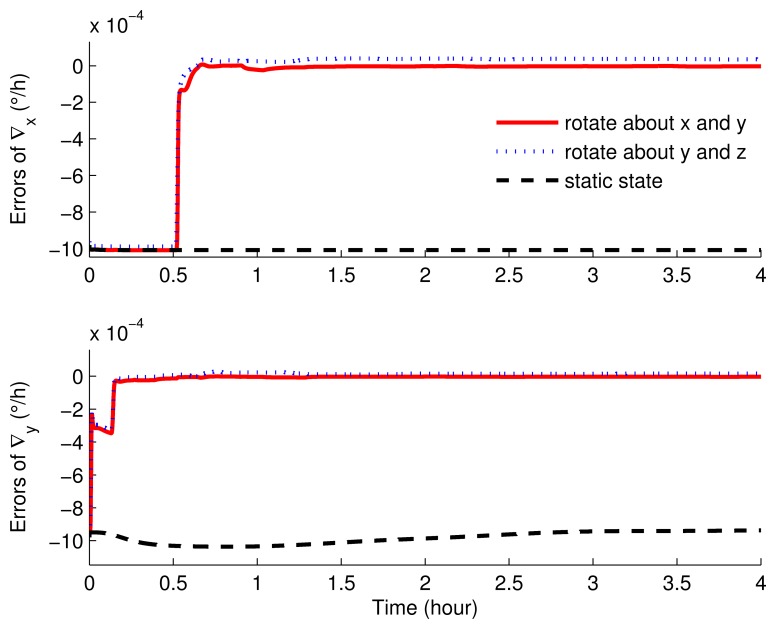
The estimated errors of the horizontal accelerometer biases of the rotation and static simulations.

**Figure 10.**
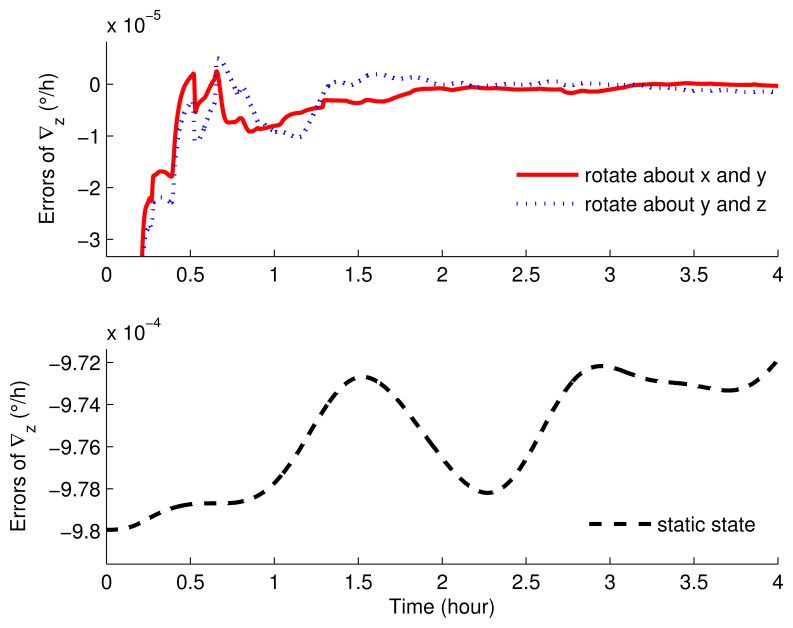
The estimated errors of the azimuth accelerometer biases of the rotation and static simulations.

The parameter settings of the simulations are shown in Table 1. Figures 5, 6, 7, 8, 9 and 10 present the comparisons of the simulation results.

The correlation error curves of the misalignment angles of the three simulations are shown in Figures 5 and 6. When the IMU was set as stationary, the misalignment errors were relatively large. That is, in stationary mode, the misalignment angles cannot be estimated, while those in the rotating simulations can be estimated readily. As can be seen, in rotating simulations, the horizontal angle errors were almost similar, except that the curves had more waves when the IMU rotated about the *y* axis and *z* axis. Regarding the azimuth angle error, the simulation with the eight-position scheme rotating about the *x* axis and *y* axis can obtain a smaller estimated error.

In Figures 7 and 8, the error curves of the gyroscope drifts were compared. As shown in these figures, in stationary mode, only *ε_y_* can be estimated, but with poor accuracy. The error of *ε_y_* still had about 0.002°/*h* left. Additionally, *ε_z_* was not restrained in 4 h, while *ε_x_* was not estimated at all. On the other hand, compared with the results of the rotary simulations, the performances with the eight-position scheme rotating around the *x* axis and *y* axis were much better. With this scheme, the horizontal gyroscope drifts were convergent and steady within 1 h, while the azimuth gyroscope drift was convergent in 1.5 h. What is more, the convergence rate and the accuracy were both superior to the other rotating simulation.

The estimated results of the accelerometer biases are as in Figures 9 and 10, which clearly show that, when the IMU was in stationary mode, ∇*_x_*, ∇*_y_* and ∇*_z_* cannot be estimated at all. With rotary simulations, the estimated results were approximately the same. Although the bias curves were all convergent within one hour, the superiority still existed in the scheme rotating around the *x* axis and *y* axis.

On the basis of the simulation results and analyses, firstly, if the IMU was rotating, a better estimation of the inertial sensor errors can be reached compared with the ones when the IMU was in stationary mode. Secondly, between these two rotating simulations, with the eight-position initial alignment and self-calibration scheme rotating about the *x* axis and *y* axis, the estimations of the inertial errors, including misalignment angles, gyroscope drifts and accelerometer biases, were much better. Therefore, the superiority and effectiveness of the rotating scheme were demonstrated evidently. Further, the feasibility of the design principle proposed in this paper was also checked at the same time.

## 5. Experiments and Results

In this section, experiments were carried on to further verify the superiority of the new eight-position rotary scheme. The SINS made by our lab and the SGT-3 turntable made by AVIC-precision 303 were used, the performance parameters of which are listed in Table 2.

**Figure 11.**
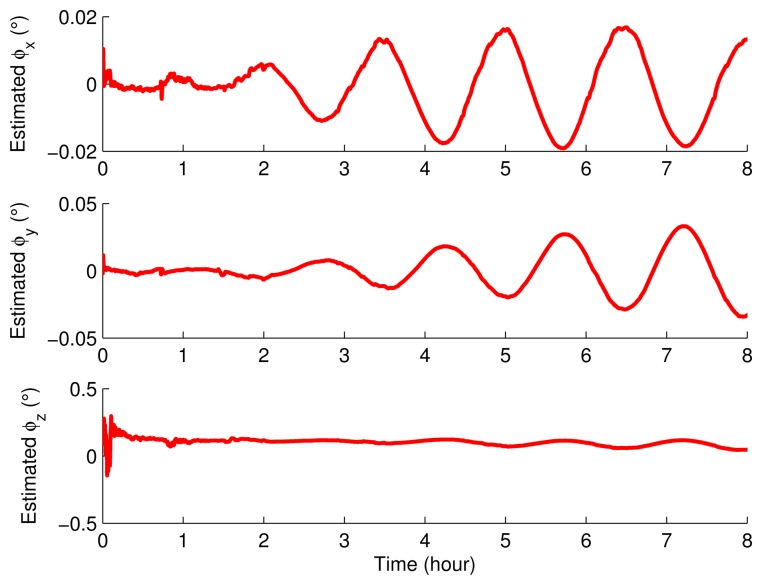
The estimated misalignment angles.

**Figure 12.**
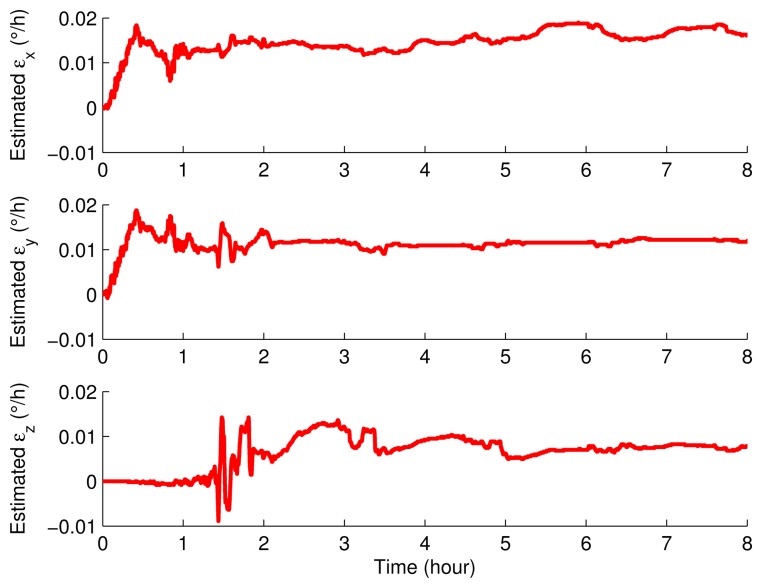
The estimated gyroscope drifts.

**Figure 13.**
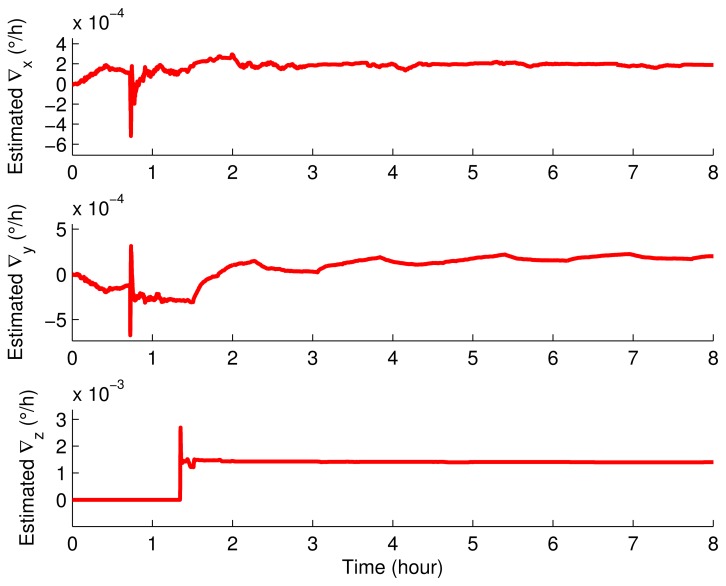
The estimated accelerometer biases.

The IMU remained static about 0.7 h and then rotated according to the rotary scheme proposed in Section 3. The inertial sensor errors and the misalignment angles were estimated by using the Kalman filter, and the results are shown in Figures 11, 12 and 13.

**Figure 14.**
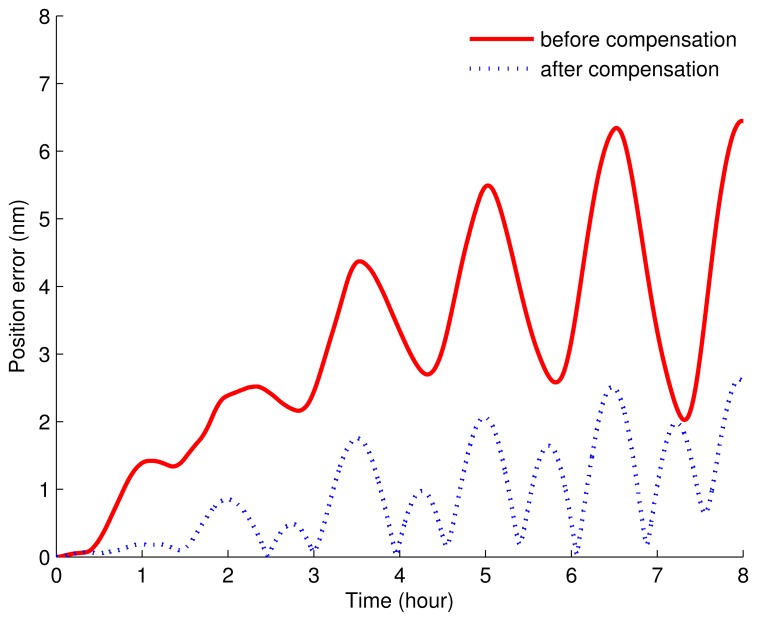
The curves of the position error before and after compensation.

It can be seen obviously from Figures 11, 12 and 13 that the estimated curves were all stable in 4 h. Thus, in order to verify the estimated results, the estimated results at 4 h were compensated for and the position errors before and after the compensation were compared, as shown in Figure 14.

From Figure 14, we can see that, before compensation, the position error was about 7 nm, while after compensation, the position error was about 3 nm. Therefore, with the new rotary scheme proposed in this paper, the misalignment angles, the gyroscopic drifts and the accelerometer zero-biases can be estimated effectively. Further, the correctness and availability of the new rotary scheme were also verified.

## 6. Conclusions

In this manuscript, the design principle of a rotating scheme in a dual-axis rotary SINS was proposed in order to improve the slow convergence speed and low estimation accuracy of inertial errors during the initial alignment and self-calibration. On the basis of this principle, a new eight-position rotating scheme was designed. A static simulation and another rotating simulation were used as comparisons, and the only difference between these two rotating simulations was that they have different rotating axes. In these rotary simulations, the IMU periodically rotates about the rotating axes to enhance the observability of the SINS. To further verify and compare, numerical simulations and experiments were carried out. The simulation results showed that better performance can be achieved when the IMU was rotating than when the IMU was static. Although the errors of the inertial sensors and the misalignment angles can effectively be estimated in three rotating simulations, the eight-position rotating scheme rotating about the *x* axis and *y* axis had the best performance. Furthermore, the experiment results showed that the position accuracy of the whole SINS can be increased significantly with this new initial alignment and self-calibration rotating scheme. However, the effect of the scale factor error was not taken into account in this study and will further be studied as one of the future tasks.

## Figures and Tables

**Table 1.** Simulation parameters.

**Parameters**	**Parameter Settings**
angular rate of rotation: *υ_r_*	5 (°/s)
angular acceleration of rotation: *a_r_*	2.5 (°/s^2^)
residence time: *t_stop_*	500 *s*
constant drifts of gyroscopes: *ε_x_* = *ε_y_* = *ε_z_*	0.01 (°/*h*)
zero-biases of accelerometers: ∇*_x_* = ∇*_y_* = ∇*_z_*	9.8 × 10^×4^ *m*/*s*^2^
noises of gyroscopes: *ω_ε_x__* = *ω_ε_y__* = *ω_ε_z__*	0.001 (°/*h*)
noises of accelerometers: *ω*_∇*_x_*_ = *ω*_∇*_y_*_ = *ω*_∇*_z_*_	9.8 × 10^−5^ *m*/*s*^2^
initial errors of horizontal misalignment angles: *φ_x_* = *φ_y_*	0.01°
initial error of azimuth misalignment angle: *φ_z_*	0.05°
initial longitude: *L*_0_	126.67°
initial latitude: λ_0_	45.78°

**Table 2.** Performance parameters of the turntable.

**Parameters**	**Parameter Settings**
load	20∼150 Kg
angular rate of rotation	0.0001∼1000 (°/*s*)
maximum of rotating angular acceleration	4000 (∼/*s*^2^)
positional accuracy	±3″
angular accuracy	±3″
